# Comparative performance of the novel, point-of-care Pluslife Mini Dock *Dirofilaria immitis/Dirofilaria repens* detection test with the modified Knott’s test in dogs

**DOI:** 10.1186/s13071-026-07401-5

**Published:** 2026-05-09

**Authors:** Tiana L. Sanders, Alexa Starnes, Maureen A. Kelly, Pabasara Weerarathne, Guilherme G. Verocai

**Affiliations:** Department of Veterinary Pathobiology, College of Veterinary Medicine and Biomedical Sciences, Texas A&M University, College Station, TX USA

**Keywords:** Heartworm disease, Microfilariae detection test, Molecular diagnostics, Pluslife Mini Dock *Dirofilaria immitis/Dirofilaria repens* point of care, RNase hybridization-assisted amplification

## Abstract

**Background:**

The current American Heartworm Society guidelines recommend the concomitant use of an antigen detection test and a microfilariae detection test (MFDT) for diagnosing heartworm infection in dogs. The modified Knott’s (MK) test is the preferred MFDT for determining the morphological characteristics of *Dirofilaria immitis* microfilariae, but it requires extensive microscopy training and can be time-consuming in a clinical setting. The Pluslife Mini Dock is a point-of-care diagnostic platform that uses RNase HII-assisted amplification (RHAM) to eliminate the need for DNA extraction, with results available within 30 min. This study aimed to assess the performance of the Pluslife Mini Dock duplex *Dirofilaria immitis/Dirofilaria repens* assay in dog blood compared with the MK.

**Methods:**

Archival, frozen whole-blood samples collected from 250 dogs at shelters in central Texas, USA, were used. Samples were subjected to the MK on the day of collection and stored at 2°C until further processing. The samples were then thawed and subjected to the Pluslife Mini Dock *D. immitis/D. repens* duplex assay. The results were analyzed using Cohen’s kappa coefficient and McNemar’s Chi-squared test.

**Results:**

Overall, 93.6% of results matched between the two tests; however, the Pluslife assay detected a higher proportion of *D*. *immitis*-positive samples (32.4%; 81/250) than the MK (30.0%; 75/250). There was no statistical significance between tests (*p* = 0.2113). Cohen’s kappa statistic indicated almost perfect agreement between the two tests (0.85). Additionally, *Acanthocheilonema reconditum* was detected in 11 samples in the MK test, without generating false-positive results with the Pluslife assay, indicating its specificity.

**Conclusions:**

Our data suggest that the Pluslife Mini Dock *D. immitis/D. repens* duplex assay provides a novel diagnostic platform and is a suitable option for point-of-care MFDT.

**Graphical Abstract:**

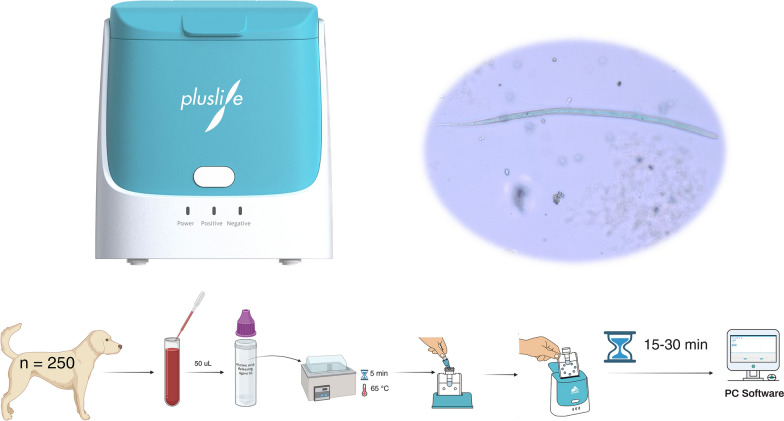

**Supplementary Information:**

The online version contains supplementary material available at 10.1186/s13071-026-07401-5.

## Background

Canine heartworm disease is caused by the filarial nematode *Dirofilaria immitis*, whose infection impacts quality of life and, if left untreated, can be fatal [1]. Adult worms reside primarily in the host's pulmonary vasculature, where they can live for up to 5 years, during which sexually mature adults produce microfilariae that circulate in the bloodstream [1-4]. The pathology caused by adult worms includes fibrosis of the endothelial lining, pulmonary hypertension, and right-sided heart failure; in severe cases, caval syndrome can occur [1, 2]. Caval syndrome is characterized by a sudden blockage of blood flow caused by the retrograde migration of a mass of adult worms from the pulmonary artery into the right ventricle, right atrium, and, in severe cases, the cranial vena cava [4, 5]. *Dirofilaria repens* is a zoonotic filarial nematode with cases primarily occurring in the Old World. *Dirofilaria repens* differs from *D. immitis* in that it is a cutaneous-dwelling filarial nematode [17]. While *D. repens* is not considered endemic to the USA, where this study was conducted, cases have been reported or suspected [18–21].

The current guidelines of the American Heartworm Society (AHS) recommend using both an antigen detection test and a microfilariae detection test (MFDT) to confirm infection and diagnose heartworm disease [2]. Various microscopy-based microfilariae detection tests are commonly used in clinical settings and reference diagnostic laboratories, with variable performance among tests [2, 30]. The modified Knott’s (MK) test is a concentration technique that kills and stains microfilariae for morphological identification and is considered more sensitive and specific than other methods, such as the direct smear, making it the AHS-recommended MFDT [2–4]. Moreover, MK may generate both qualitative and quantitative results, with the latter often recommended in cases of suspected macrocyclic lactone (ML) resistance (e.g., microfilariae suppression test) [31]. However, the MK test requires expertise in morphological identification to discriminate at the species level between *D. immitis* and other filarial nematodes that infect dogs, such as *D. repens* and *Acanthocheilonema reconditum* [14]*.* Furthermore, because the MK test is relatively time-consuming and not feasible to perform in a clinic, tests are typically sent to reference laboratories, where processing time may delay results.

More recently, various researchers have focused on the validation and optimization of molecular techniques as alternatives to microscopy-based microfilariae detection tests [14, 30, 35, 37]. Isothermal amplification technologies, such as loop-mediated isothermal amplification (LAMP), provide an alternative diagnostic platform and have been used to detect a variety of pathogens [6-9]. LAMP technology enables specific amplification of a target under isothermal conditions without the need for DNA extraction, with results available in a short period of time (e.g., 30 min). Furthermore, other nucleic acid detection methods, such as real-time PCR (qPCR), require extensive training and expensive equipment, and they may be less accessible than LAMP assays [10, 11, 14]. LAMP assays do not require a thermocycler, as they operate at a constant temperature and can be performed with a simple heat block [38, 39]. However, a limitation of LAMP-based assays is that they can produce non-specific amplification, leading to false negatives [12]. To combat this, a novel amplification method, RNase HII-assisted amplification (RHAM), was developed and consists of a LAMP assay with the addition of an RNase HII fluorescent probe for target signal visualization [10, 11]. RHAM technology includes an internal positive control to ensure sample integrity and can incorporate multiple targets in a single test. Furthermore, RHAM technology has been used to detect other pathogens such as SARS-CoV-2 and African swine fever virus [10–13]. Therefore, the objective of the present study was to compare the performance of a commercially available test platform, the Pluslife Mini Dock *D. immitis*/*D. repens* assay, with the results of the microscopy-based MK test as an alternative microfilariae detection test method utilizing canine blood.

## Methods

The aim of this study was achieved by using archival, frozen whole-blood samples from shelter dogs residing in Brazos County, Texas, USA, collected from 2020 to 2022 under an approved Animal Care and Use Protocol (2022-0261) [14]. The inclusion criteria were dogs > 6 months old and no recent history of ML use [14]. Briefly, whole blood was collected in EDTA tubes and stored at 2°C until the MK was performed later that same day. Following processing, each sample was subjected to the MK test as described by Negron et al. [14], with results reported both qualitatively and quantitatively (mf/ml). Any remaining samples were stored at − 20°C until further analysis.

The Pluslife assay was initiated by thawing each blood sample on ice, followed by a brief vortex, and then adding 50 μl to the Nucleic Acid Release Tube (Additional File 1: Fig S1). The tube was then inverted 8–10 times and incubated in a water bath at 65°C for 5 min. After incubation, the lysate was added to the Pluslife *D. immitis/D. repens* Nucleic Acid Reaction Card (patented by Guangzhou Pluslife Biotech, Guangzhou, China). The reaction card was left to stand for 15 s. Following this, pressure was applied to the domed cap; the reaction card was shaken for 10 s and then inserted into the Pluslife Integrated Nucleic Acid Testing Device. The process was then initiated by pressing the start button with testing conducted within 30 min. An example of the Pluslife workflow, a positive result for *D. immitis*, and a negative result for *D. repens* is shown in Additional File 1: Fig S1. The Pluslife software displays a timer, and the time it took for samples to turn positive was recorded. The software provides either a positive or negative result, along with the amplification curve, once each sample has been run (Additional File 1: Fig S1). All results were visually assessed by reviewing the software-provided amplification curves to further confirm the correct assignment of positive and negative results for both *D. immitis* and *D. repens*. Any amplification curves that varied from the positive example the software provided were forwarded to technical support. Furthermore, because *D. repens* is rare in the USA, any sample positive for *D. repens* was tested by PCR using the previously described primers and cycling conditions [22].

**Fig. 1:**
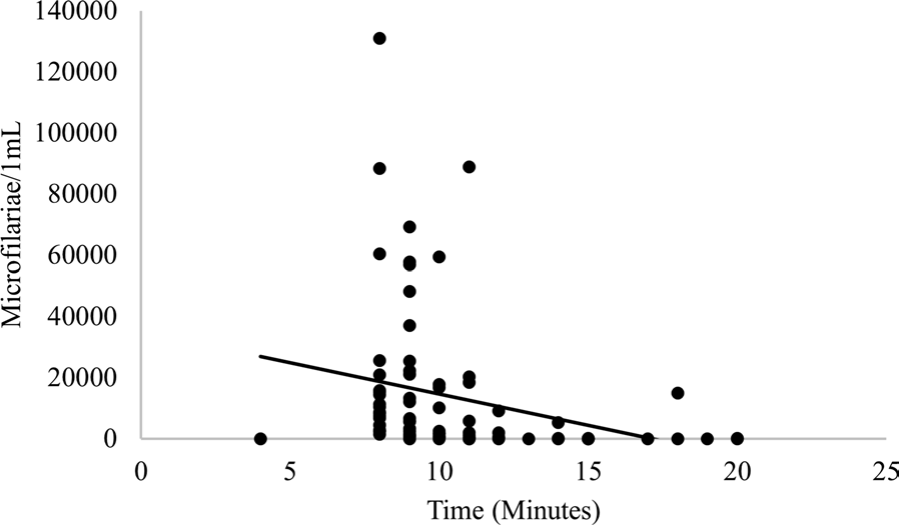
Scatter plot of modified Knott’s microfilariae counts versus Pluslife assay time to positivity in minutes

To determine the agreement between the MK and Pluslife assay, Cohen’s kappa coefficient (κ) was calculated [15]. Kappa values were then interpreted based on the following thresholds: slight (< 0.20), fair (0.21–0.40), moderate (0.41–0.60), substantial (0.61–0.80), and almost perfect (0.81–1.00) [15]. A McNemar’s Chi-square test was performed to determine the comparative diagnostic performance between the two assays, with *p* < 0.05 considered statistically significant. Pearson’s correlation was performed to determine whether the time to positivity on the Pluslife assay was correlated with the microfilariae count determined from the MK (Fig. 1). All statistical analyses were conducted using RStudio version 2025.05.0 + 496.

## Results

Overall, the Pluslife assay identified 32.4% (*n* = 81/250) of samples as *D. immitis*-positive, whereas the MK test identified 30.0% (*n* = 75/250) *D. immitis* [14]. Among the 250 paired results, 70 samples were positive for both assays, and 164 were negative in both assays, yielding an overall agreement of 93.6%. Additionally, 16 samples were positive in only one test. Initially, the Pluslife Mini Dock duplex *D. immitis/D. repens* assay co-detected *D. immitis* and *D. repens* in a single sample, but following visual assessment of the amplification curve, it was considered a false positive for *D. repens*. Molecular analysis further confirmed that *D. repens* was not present in the sample. Cohen’s kappa statistic showed an almost perfect agreement between the tests (0.85) (Table 1). McNemar’s test indicated there was no statistical difference between the MK and Pluslife assay (*p* = 0.2113) (Table 1).

**Table 1:** Summary statistics for the comparison of the Pluslife assay and the modified Knott’s test

Test comparison	a	b	c	d	Cohen’s *κ*	Agreement	*χ*^2^	df	*p-value*
PL vs MK	70	11	5	164	0.85	Almost Perfect	1.5625	1	0.2113

*PL* Pluslife assay, *MK* modified Knott’s test, *κ* Cohen’s kappa, *χ*^2^ McNemar’s Chi-square statistic, *df* degrees of freedom

Samples testing positive for *D. immitis* via the Pluslife assay generated positive signals in an average of 10.5 min ± 3.2 min from the start of the reaction (range: 4–20 min). There was a weak negative correlation between microfilariae counts (average: 15,502.6 mf/ml ± 24,697.7; range: 103.5–130,927.5) and the time to positivity in the assay for *D. immitis*, indicating that higher microfilariae counts are associated with shorter time to positivity (*r* = − 0.26, 95% CI − 0.45 to − 0.05, *p* = 0.017). In addition, *A. reconditum* microfilariae were detected in 11 samples using the MK test [14], but none of these generated false-positive results on the Pluslife assay, further supporting its analytical specificity.

## Discussion

The findings of this study demonstrate that the RHAM-based Pluslife Mini Dock duplex *D. immitis/D. repens* assay performs comparably to the MK test in qualitative diagnosis of *D. immitis* microfilariae in canine blood. Therefore, it provides an efficient alternative to established microfilariae detection tests for use in clinical and diagnostic settings without the need for classical parasitology expertise. The Pluslife assay detected six additional samples that were not positive with the MK. This could potentially be due to low microfilaremia at the time of blood collection. Although MK is the AHS-recommended MFDT and consistently outperforms other microscopy-based tests, veterinarians in clinics may opt for other microfilariae-detection tests, such as the direct smear, because of their lower cost, faster results, and minimal equipment requirements [2, 30]. Therefore, a study based on a direct comparison of the Pluslife Mini Dock duplex *D. immitis/D. repens* with other widely used MFDTs, in particular direct smears, is warranted.

The current AHS guidelines for diagnosing *D. immitis* include the use of both an antigen detection test and an MFDT, which allows for species-level identification and may yield quantitative results [2]. However, there are multiple instances in which the MFDT yields discordant results compared with antigen detection tests in occult infections. This poses a challenge for clinicians, who must then perform additional diagnostic tests that may delay results and the potential for medical intervention. For example, instances in which microfilariae are detected but no antigen is found may be due to other filarial nematodes of dogs, such as *D. repens* or *A. reconditum*, the formation of antigen-antibody immune complexes, or adult worms that may have died or been killed while microfilariae remain [2, 14, 23]*.* In the presence of another filarial nematode, the ability to distinguish microfilariae morphologically is crucial but may be difficult if less-sensitive MFDTs, such as the direct smear, are used [24]. Therefore, a reliable, user-friendly, and efficient MFDT is paramount.

The ability of an MFDT to quantify microfilariae is clinically important for several reasons. In suspected cases of ML resistance, a microfilariae suppression test may be performed and requires quantifying microfilariae before and after ML administration to provide a presumptive diagnosis [31]. Additionally, while adult heartworms cause extensive cardiovascular pathology, including endothelial damage, fibrosis, and obstruction, microfilariae cause independent pathological effects such as inflammation and glomerulonephritis [1–3, 16, 17, 33, 34]. The negative correlation between time to positivity on the Pluslife assay and microfilariae count on the MK indicates that a higher microfilariae count may produce a faster positive result (*r* = − 0.26, 95% CI − 0.45 to − 0.05, *p* = 0.017). Nevertheless, the weak correlation with MK suggests a poor quantitative performance of Pluslife; however, further validation is required to determine the quantitative potential of this novel test.

Molecular tests for detecting *D. immitis* have become widely available to veterinarians, offering greater sensitivity and specificity [30, 35, 37]. Recent studies have shown that MK results are comparable to those obtained with conventional PCR but underperform relative to qPCR [14, 30, 32]. Currently, we do not know how the Pluslife Mini Dock duplex *D. immitis/D. repens* performs compared with other molecular tests, and this should be further investigated. Nevertheless, the compact and portable design of the Pluslife Mini Dock enables easy implementation in clinical settings, in contrast to most currently available molecular assays, which are typically intended for use in reference diagnostic laboratories.

The ability to multiplex molecular assays to detect multiple targets within a single test reduces the need for microscopy training and expertise in morphological characteristics and may enable the acquisition of more comprehensive test results from a single sample. Previously published molecular assays that utilize next-generation sequencing and/or qPCR assays have been applied to detect a range of gastrointestinal parasites, vector-borne pathogens, and filarial nematodes of veterinary importance [28, 29, 35, 36]. Although the Pluslife *D. immitis*/*D. repens* assay currently has two targets plus an internal control, the Pluslife Nucleic Acid Reaction Card supports multiplexing up to seven targets. This could include other blood- or skin-dwelling filarial nematodes that infect companion animals, such as *A. reconditum*, *Onchocerca lupi, Cercopithifilaria bainae*, and other species within the genus *Dirofilaria* [23, 25, 26] or other clinically relevant hemoprotozoans [27, 28].

Whereas this study provides insight into a novel point-of-care test, some limitations should be acknowledged. In this preliminary study, the archived samples had undergone multiple freeze-thaw cycles and may have experienced some degree of degradation. Notably, the manufacturers recommend testing fresh whole blood to ensure the highest detection accuracy. Therefore, DNA degradation could have affected the Pluslife assay results, yielding lower positivity than in fresh samples. Future studies should utilize fresh whole-blood samples to mitigate this potential limitation. Furthermore, as the AHS [2] recommends using both an antigen detection test and MFDT to diagnose *D. immitis*, it would be valuable to compare the performance of the Pluslife assay when paired with an antigen detection test and MK testing. This, in turn, will help determine the overall detection in real time.

## Conclusions

This study compared the performance of the novel point-of-care Pluslife Mini Dock Duplex *D. immitis/D. repens* test with the modified Knott’s test for the detection of *Dirofilaria immitis.* The Pluslife assay detected a higher proportion (*n* = 81; 32.4%) than the MK test (*n* = 75; 30%), with almost perfect agreement (*κ* = 0.85). These results demonstrate that the Pluslife assay is a suitable MFDT that can be conveniently implemented for in-clinic use and provides results within 30 min.

## Supplementary Information

Additional file 1: Figure S1: Illustration of the workflow of the Pluslife *Dirofilaria immitis/D. repens* assay and an example of results generated by the Pluslife software.

## Data Availability

Data supporting the main conclusions of this study are included in the manuscript.

## Abbreviations

AHS: American Heartworm Society
LAMP: Loop-mediated isothermal amplification
ML: Macrocyclic lactone
MFDT: Microfilariae detection test
MK: Modified Knott’s
RHAM: RNase HII-assisted amplification
