# Supplementary figures and images for: Comparative performance of the novel, point-of-care Pluslife Mini Dock Dirofilaria immitis/Dirofilaria repens detection test with the modified Knott’s test in dogs
Source: Parasit Vectors. 2026 May 9;19:270. doi: 10.1186/s13071-026-07401-5 (PMC13326338; doi:10.1186/s13071-026-07401-5)

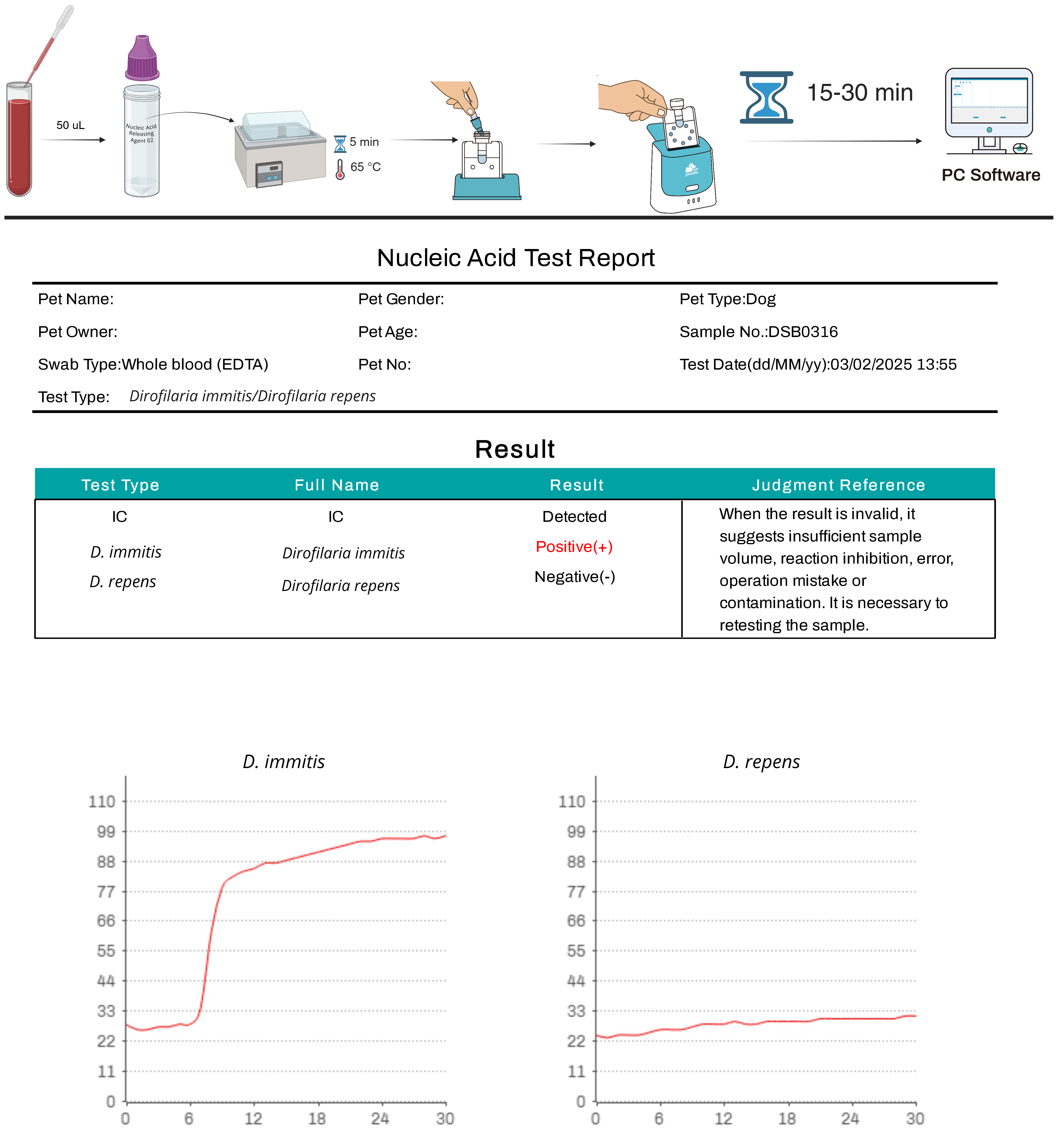

Supplement: Supplementary file 1 — Additional file 1: Figure S1: Illustration of the workflow of the Pluslife Dirofilaria immitis/D. repens assay and an example of results generated by the Pluslife software. [file 13071_2026_7401_MOESM1_ESM.png]
